# Interactive knowledge discovery and data mining on genomic expression data with numeric formal concept analysis

**DOI:** 10.1186/s12859-016-1234-z

**Published:** 2016-09-15

**Authors:** Jose M González-Calabozo, Francisco J Valverde-Albacete, Carmen Peláez-Moreno

**Affiliations:** Department of Signal Theory and Communications, University Carlos III Madrid, Avda. Universidad, 30, Leganés (Madrid), Spain

**Keywords:** Biclustering, Gene expression data, Formal concept analysis, Exploratory data analysis, Gene set enrichment, Knowledged discovery, Data mining

## Abstract

**Background:**

Gene Expression Data (GED) analysis poses a great challenge to the scientific community that can be framed into the Knowledge Discovery in Databases (KDD) and Data Mining (DM) paradigm. Biclustering has emerged as the machine learning method of choice to solve this task, but its unsupervised nature makes result assessment problematic. This is often addressed by means of Gene Set Enrichment Analysis (GSEA).

**Results:**

We put forward a framework in which GED analysis is understood as an Exploratory Data Analysis (EDA) process where we provide support for continuous human interaction with data aiming at improving the step of hypothesis abduction and assessment. We focus on the adaptation to human cognition of data interpretation and visualization of the output of EDA.

First, we give a proper theoretical background to bi-clustering using Lattice Theory and provide a set of analysis tools revolving around $\mathcal {K}$-Formal Concept Analysis ($\mathcal {K}$-FCA), a lattice-theoretic unsupervised learning technique for real-valued matrices.

By using different kinds of cost structures to quantify expression we obtain different sequences of hierarchical bi-clusterings for gene under- and over-expression using thresholds. Consequently, we provide a method with interleaved analysis steps and visualization devices so that the sequences of lattices for a particular experiment summarize the researcher’s vision of the data. This also allows us to define measures of persistence and robustness of biclusters to assess them.

Second, the resulting biclusters are used to index external omics databases—for instance, Gene Ontology (GO)—thus offering a new way of accessing publicly available resources. This provides different flavors of gene set enrichment against which to assess the biclusters, by obtaining their *p*-values according to the terminology of those resources.

We illustrate the exploration procedure on a real data example confirming results previously published.

**Conclusions:**

The GED analysis problem gets transformed into the exploration of a sequence of lattices enabling the visualization of the hierarchical structure of the biclusters with a certain degree of granularity. The ability of FCA-based bi-clustering methods to index external databases such as GO allows us to obtain a quality measure of the biclusters, to observe the evolution of a gene throughout the different biclusters it appears in, to look for relevant biclusters—by observing their genes and what their persistence is—to infer, for instance, hypotheses on their function.

**Electronic supplementary material:**

The online version of this article (doi:10.1186/s12859-016-1234-z) contains supplementary material, which is available to authorized users.

## Background

In the present framework to analyze Gene Expression Data (GED)—be it on microarray expression profiles [1] or the more recent and higher quality profiles obtained by the so-called *Next Generation Sequencing (NGS)* [2–4]— the data are eventually represented as a gene expression matrix $X\in \mathbb {R}^{m\times {n}}$ with *m* rows representing genes and *n* columns representing each an empirical sample or condition.

Initially, clustering genes by gene expression similarity was the technique of choice to try to induce what proteins are being synthesized under what conditions in different samples of cells. The inception of insights about gene behavior are then facilitated by these groupings.

However, comparisons of different clustering algorithms applied to gene expression [5–7] did not lead to clear conclusions about their performance since the results are highly depending on the data analyzed. The unsupervised nature of GED analysis problem also prevents a systematic evaluation of algorithms, since in most situations there is no previously defined ground-truth.

Also, the idea of non-overlapping clusters or partitional clusterings might not be adequate, since overlapping functional relations between genes or similarities of conditions are obscured in such clusterings. Further technical difficulties are the need fora priori choosing a distance metrics and, for some popular methods like *k-means* [8] or *self-organizing maps* (SOM) [9] an a priori knowledge of the number of clusters.

Some of these problems can be solved by Exploratory Data Analysis (EDA) [10], but basic clustering techniques lack the interactivity and flexibility capabilities desirable in a tool design for exploration. *Hierarchical clustering* is an alternative for solving the exploratory difficulties producing a *dendrogram* that not only identifies the clusters but also the similarity between them, and allows a certain overlap in the explored clusters, though not on the finally chosen ones. Its lack of robustness is its main drawback [11].

Another limitation of clustering is that the domain of the analysis, i.e. whether to group genes or empirical samples, also needs to be decided a priori and either one or the other may be applied. *Bi-clustering* [12], also known as *co-clustering* or *two-mode clustering*, provides us with the possibility of simultaneously performing both combining genes and sample groupings. The intuition is that the transcription of genes sampled under differing expression conditions can be modeled as the aggregation of the effect of different biologically-plausible phenomena, having their computational correlates in *biclusters*, pairs of sets of indices into the genes and conditions in a gene expression matrix. In the last few years, bi-clustering has emerged as the unsupervised method of choice for GED [1, 13, 14].

For instance, the authors of the Iterative Signature Algorithm (ISA) [15, 16] posited the existence of *transcription modules*—these being the coupled sets of conditions and genes— whereby the expression level of a particular gene *g* in a condition *c* is an aggregation of the discretized activities of all the transcription modules *g* and *c* belong to:

1 $$\begin{array}{*{20}l} X_{gc} = \left(P A^{\mathrm{T}}\right)_{gc} = \sum_{k=1}^{p} P_{gk} A_{ck} \end{array} $$

where *k*∈{1,…,*p*} ranges over the possible transcription modules or factors, *P*∈{0,1}^*m*×*p*^ is a matrix each of whose columns *P*_·*k*_∈{0,1}^*m*^ is a promoter vector describing if transcription factor *k* activates each gene and *A*∈{0,1}^*n*×*p*^ is a matrix each of whose columns *A*_·*k*_∈{0,1}^*n*^ is a vector describing whether the transcription factor *k* is active in condition *c*. Both of these kinds of vectors are sparse: ISA first discretizes the gene-expression matrix *X* into *I* by means of gene- and condition-relative thresholds *φ*_*g*_ and *φ*_*c*_, respectively, and then carries out the bicluster analysis. Note, also, the relationship of such models to Non-negative Matrix Factorization (NMF).

A prevalent phenomenon in gene expression measurement is that due to experimental variation, thermodynamical fluctuations and other uncontrolled conditions, measurements are quite noisy, and often include a number of outliers, the advantages of NGS over conventional microarrays in this respect notwithstanding. For this reason, the generative multiplicative model for *K* biclusters used in e.g. FABIA [14] includes an error model. The generic form of this model is:

2 $$\begin{array}{*{20}l} X = \sum_{k=1}^{p} \lambda_{k} z_{k}^{\mathrm{T}} + \Psi \end{array} $$

where the *λ*_*k*_ are the prototype gene expression vectors containing zeros for genes not participating in the bicluster, *z*_*k*_ are the vectors containing zeros for conditions not participating in the bicluster, and *Ψ* is an error matrix, to be minimized. The bicluster itself adopts the form of a subblock of the matrix whose rows and columns are approximately proportional, as measured by their scalar product.

A desirable feature in these models is to allow *bicluster overlapping* to reflect the fact that a particular gene can participate in different biological processes (modules, functions) for different conditions.

It was already noticed in [17] that overlapping allows the possibility of some biclusters being “included” within others and used a hierarchical depiction of this order to suggest the unfolding of finer and finer structure with the evolution of a threshold parameters.

Regardless of the bi-clustering method adopted, measurement variability, the dual roles of genes and conditions and the sheer number of relations and factors to be considered hinder the human analyst’s intuition to be brought to bear in the process of GED analysis. Therefore, it might profit from Human-Computer Interaction (HCI) for Knowledge Discovery in Databases and Data Mining (KDD&DM) [18].

KDD&DM is a conceptual framework including a set of *desiderata, tools and practices* for the analysis of data, that encourages complementing traditional Confirmatory Data Analysis (CDA) with human interaction to perfect the step of hypothesis abduction. For this purpose, the interpretation and visualization of data and the results of the data mining process have to be adapted to human cognition. Examples of such effort can be found in [19] where visualization requirements based on domain experts’ interviews are enumerated, or [16] where exploratory analysis in the guise of a hierarchical diagram of models is depicted and co-indexed with other exploratory plots, like heatmaps.

Exploratory Data Analysis (EDA) is the proper statistical framework to carry out KDD&DM. Though gaining momentum in several fields, this paradigm—to be contrasted to CDA—shows a number of challenges as applied to the analysis of GED: having a trustworthy technique to measure gene expression, supporting the induction process, and evaluating the result of induction. Further insights into this issue will be reviewed in Discussion.

Formal Concept Analysis (FCA, [20]) can be conceptualized as an unsupervised, non-partitional hierarchical co-clustering algorithm for binary input tables based in lattice theory. Application of these techniques for GED can be found for instance in [21] that shows a method to identify biomarkers in breast cancer, in [22] where it is employed to find a list of genes for inclusion into a partially known basic gene network, in [23] that describes two different but mathematically equivalent FCA methods to cluster genes or in [24] for consensus clustering of multi-experiment expression data.

FCA supports EDA both at a theoretical level, by conceptualizing exploration as an embedding in *landscapes of knowledge* [25, 26], and practically, by providing visual, interactive diagram exploration tools that depict and describe the relation between genes and conditions in a condensed, yet highly intuitive form.

Unfortunately, FCA is ill-adapted to dealing with GED *numerical* data. Rather, it has to use a preprocessing technique, *scaling*, whereby numerical data tables are transformed into binary ones.

In this paper, we demonstrate an exploratory method for GED based on $\mathcal {K}$-Formal Concept Analysis ($\mathcal {K}$-FCA, [27]), a generalization of FCA where entries in data tables may be non-binary numbers taking values in a scale $\mathcal {K}$ designed to convey statistical information between genes and conditions, in the form of a lattice of bi-clusters.

This data-driven method does not require previous knowledge of the distribution of the data, nor is it necessary to define any distance metrics or give an estimation of the number of clusters, and it provides a browsable representation of the hierarchy of overlapping biclusters in the form of a lattice at each chosen level of resolution.

The proposal is well-founded and completes the “landscapes of knowledge” metaphor providing a sound basis for EDA over non-boolean data with FCA that enables exploring the data at different resolutions [28]. As we sweep over those resolutions, the output is a sequence of gene expression lattices, where the researcher can see how clusters evolve, enabling a more detailed and less rigid understanding of the behavior some genes may share under different conditions. To improve this understanding, the gene lattices are used as an indexing mechanism onto external ontologies, thus providing a flavor of Gene Set Enrichment.

In this way, we introduce an expressive bi-clustering algorithm—$\mathcal {K}$-FCA—coupled with its natural visualization method—a sequence of order diagrams or lattices—adapted for GED analysis in a suitable EDA framework, intended as an aid for decision and research by experts.

We also provide for open access WebGeneKFCA [29], the prototype tool employed for the analysis, at http://webgenekfca.com. As explained in Interfacing with gene ontologies, to support the exploratory process, the tool offers a brief description for each gene selected from the biclusters. This description has references to the NCBI gene database^1^ to ease the access to the latest online description of that gene. There are also references to each of the Gene Ontologies^2^ the gene belongs to, with one link to each ontology description including *p-values* (see Exploration: gene set analysis). Finally WebGeneKFCA shows whether the gene is known to belong to a pathway and provides a link to the KEGG pathway database^3^.

To illustrate the analysis we advocate we have carried out the analysis of some public GED and will argue for the consistency of our results with those of the paper originally describing them. The particular example of this paper can be found at https://webgenekfca.com/webgenekfca/datamatrices/7 where the reader can interactively explore the output lattices at different levels.

## Methods

### A Formal Concept Analysis primer

FCA [20] is an unsupervised biclustering algorithm for binary data based in lattice theory [30] that, apart from providing the desired biclusters of *gene* and *condition* sets establishes a *partial order* in them, usually represented as a *lattice* diagram.

Assume for the moment that GED are collected in a boolean matrix $I \in \mathbb {B}^{m \times n}$ of *m* genes (or *objects* in FCA parlance) with *n* possible samples or conditions (called *attributes* in FCA) where an object can have or not a given attribute. The triple of the set of genes *G*, conditions *C* and the relation is called a *formal context* (*G*,*C*,*I*) and it carries two polar functions: the first one can obtain all the conditions in which any set of genes *x* occur, while the second can obtain all the genes in which a set of conditions *y* occur.

3 $$\begin{array}{*{20}l} x^{\uparrow} = \left\{ c \in C \mid \forall g \in x, gIc \right\}\quad y^{\downarrow} = \left\{ g \in G \mid \forall c \in y, gIc \right\} \end{array} $$

Despite being mutually recursive, these two functions find their fixpoints in just two steps

$$\begin{array}{*{20}l} \left(\left(x^{\uparrow}\right)^{\downarrow}\right)^{\uparrow} = x^{\uparrow}\qquad \left(\left(y^{\downarrow}\right)^{\uparrow}\right)^{\downarrow} = y^{\downarrow} \end{array} $$

A *formal concept* (here, a bicluster) is the pair of an *extent* (set of genes) *a*⊆*G* and an *intent* (set of conditions) *b*∈*C* which define each other mutually.

4 $$\begin{array}{*{20}l} (a, b) \in \mathfrak{B}(G,C,I)\quad \Leftrightarrow \quad a^{\uparrow} = b\; \&\; b^{\downarrow} = a \end{array} $$

Note how formal concepts fulfill the definition of biclusters or factors in [15].

The set of formal concepts is $\mathfrak B(G,C,I)$. Formal concepts are partially ordered by the inclusion (resp. reverse inclusion) of extents (resp. intents). This means that for every two concepts *c*_1_=(*a*_1_,*b*_1_) and *c*_2_=(*a*_2_,*b*_2_) there is a concept order

5 $$\begin{array}{*{20}l}  c_{1} \leq c_{2} \Leftrightarrow a_{1} \subseteq a_{2} \Leftrightarrow b_{1} \supseteq b_{2} \end{array} $$

and therefore the set of formal concepts with this order is actually a complete lattice $\underline {\mathfrak {B}}(G,C,I)$. In this instace we call it call the *gene expression lattice*.

**Creating and reading gene expression lattices** FCA provides efficient generic procedures and tools [20] for:

1. Finding all the biclusters (*a*,*b*).
2. Finding the ordering between biclusters ≤.
3. Finding a compact set of biclusters that generate all others by a well understood method.
4. If the sets of genes or conditions are sufficiently small, drawing the lattice as an order diagram and navigate it using a point-and-click interface.

So by casting GED as formal contexts, biclusters as formal concepts and hierarchies as concept lattices we are capable of carrying out extensive exploratory analysis of GED using the FCA machinery.

**Fig. 1:**
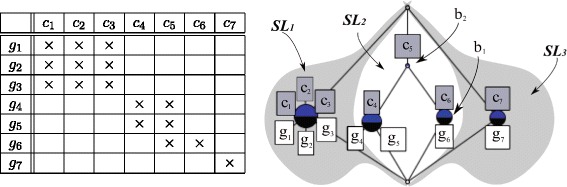
Example of a synthetic gene expression boolean matrix (*left*) and lattice (*right*). Genes are represented in *white boxes* while samples or conditions are shown inside *gray boxes*. A reduced labeling strategy is employed (see text). Adapted from [46]

Figure 1 is an example of a classical depiction of a lattice diagram (right) corresponding to a synthetic boolean matrix (left) where each of the nodes represent a concept (or bicluster). For the purpose of reading extents and intents of the lattice diagram, biclusters could be annotated graphically with a *complete labeling*, by listing for each bicluster the set of genes (white boxes) in the extent and the set of samples (gray boxes) in the intent. But since this implies repeating many times each gene and condition throughout the lattice (the biclusters are overlapping) the following, *reduced labeling* is preferred: we put the label of each condition only in the highest (most abstract) concept it appears, and the label of each gene only in the lowest (most specific) concept it appears. So conditions usually appear *just above* the corresponding concept and genes appear *just below* and each only once for the whole lattice, thus diminishing the visual clutter.

In this labeling scheme, to recover the set of genes of a particular bicluster—the *extent*—we take the union of all (white) gene labels found from the node *downwards* in the lattice. Similarly, to build the set of conditions—the intent—we take the union of all the (gray) condition labels found from the node *upwards* in the lattice. In the example, if we go from *b*_1_ downwards in the lattice collecting gene labels (below the nodes) we obtain its extent *g*_6_, and if we go upwards we find the condition labels in its intent, *c*_6_ (above *b*_1_ itself) and *c*_5_ (above *b*_2_). Thus, *b*_1_=({*g*_6_},{*c*_5_,*c*_6_}) and *b*_2_=({*g*_4_,*g*_5_,*g*_6_},{*c*_5_}) are overlapping biclusters but *b*_2_ appears above *b*_1_ (i.e. *b*_1_≤*b*_2_) because {*g*_6_}⊆{*g*_4_,*g*_5_,*g*_6_} (i.e., the set of genes in *b*_1_ are contained in the set of genes of *b*_2_) and {*c*_5_,*c*_6_}⊇{*c*_5_} (i.e. the conditions for which the genes of *b*_1_ are expressed include the conditions for which the genes of *b*_2_ are expressed).

Finally, the biclusters (or sets of biclusters) that do not have any overlappings appear in the lattice as separate sublattices (although strictly speaking they all share the top and bottom nodes). In Fig. 1 three separate sublattices can be observed: *SL*_1_ (to the left, shadowed in gray) including a single bicluster plus the top and bottom concepts; *SL*_2_ (at the center) with three biclusters plus top and bottom; and *SL*_3_ (to the rigth, also shadowed in gray), also with a single bicluster plus top and bottom. Concepts in different sublattices are incomparable except for the top and bottom. We will say that such sublattices are *adjoined or parallel sublattices* of the GED lattice.

There are many different available algorithms to visualize a Concept Lattice, each with its advantages and disadvantages, some based on square grids, others in attraction or repulsion forces, etc. A review can be found in [31]. In this paper, most of the lattices depicted are obtained with *WebGeneKFCA* that provides our own adaptations of the visualization schemes for GED (see section Lattice visualization adaptation for GED).

### From FCA to kFCA: exploring non-boolean gene expression data

The main drawback of FCA is the requirement that the gene expression quantification be boolean.

We overcome this restriction using a generalization called $\mathcal K$-FCA [27, 32, 33], where $\mathcal K$ is a type of cost adequate to measure gene expression levels. Its choice depends on the data preprocessing step and whether we want to explore gene expression for over or under-expression^4^.

$\mathcal K$-FCA introduces an extra parameter in the exploration procedure, the *threshold of existence* for biclusters to be considered: for each threshold level *φ* we may obtain a different *φ*-lattice and *φ*-concepts. For instance, for under-expression analysis this parameter $\varphi \in \mathbb R$, describes a *maximum level of expression* allowed for pairs of genes and conditions (*g*,*c*) to be considered as members of a bicluster (the *φ*-concept).

Therefore to obtain all the possible biclusters it is necessary to calculate the *sequence of lattices* defined as a function of the different *φ* threshold values. Just as a time-ordered sequence of images constitutes a movie that could be explored frame by frame using *rewind* and *forward* operations, our depiction of the *φ*-ordered sequence of lattices casts the process of *lattice exploration* as the observation of the lattices as they evolve with *φ*, as will be illustrated in section The evolution of the number of concepts with thresholding. Indeed, with this procedure the explorer can progressively choose from a coarser view of the data (with a very demanding *φ*) that only allows the most salient biclusters to appear or zoom in into finer views where this threshold is relaxed to offer a more detailed lattice with a larger number of biclusters (see section Lattice visualization adaptation for GED). Our own graphical representation of the sequence of lattices that ensures that biclusters with the same set of conditions are always plotted in the same spatial coordinates independently of *φ* and that has been specifically designed for facilitating the exploration of GED, also allows us to define a measure of the *persistence* or *robustness* of a bicluster, as will be discussed in Lattice visualization adaptation for GED.

### Lattice visualization adaptation for GED

Providing visually smooth transitions between consecutive lattice representations is the key for easing the expert process of discovering meaningful biclusters. In [29] we proposed a scheme having the distinctive feature that the biclusters with the same set of conditions are always plotted in the same position through different concept lattices^5^. This means that as the user explores the values of *ϕ* (or *φ*) she will easily see how the set of genes of each bicluster evolves, increasing or decreasing until disappearing.

In our particular example, with *n*=9 conditions, this silhouette will exhibit 10 levels or rows including the top and bottom (see Figs. 3, 4, 5 and 6). This can be observed in our example where the intents (i.e. the set of conditions) of the maximum of 9 concepts of the second row (right below the top) have only a single element: each of the conditions. The third row will be composed of up to $\binom {9}{2}~2$-combinations of the previous row concepts’ intents (i.e. all the possible pairs of conditions), the fourth, up to $ \binom {9}{3}~3$-combinations, etc.

Unlike the standard representation of lattice diagrams of Fig. 1, where genes and conditions were represented in separate white and gray boxes respectively, our adaptation for GED takes into account the fact that |*G*|≫|*C*| and substitutes the pair of boxes per node by a single one indicating the set of conditions and the number of genes belonging exclusively to that particular node, usually too large to be listed. Nonetheless, selecting the node by clicking on it provides access to the full list of genes. As in Fig. 1, to complete the list of genes of the bicluster we have to take the union of all the genes found in the labels from that node downwards.

To illustrate this, in Fig. 3 we have included the labels for every node^6^. For example, the second biggest node (the biggest is the top) represents a bicluster with only one condition (*MaleIPS*) and 14 own genes (i.e. those that are only under-expressed for that condition and no other)^7^. Also, the different rows of the lattice show the hierarchy previously explained where the second row presents the biclusters of the lattice with a single condition each, the third contains those nodes that include extensions with pairs of conditions, etc.

### Gene set enrichment with external ontologies

#### Interfacing with gene ontologies

As very early noted by Godin and collaborators [34, 35] a Formal Concept Analysis-based lattice can be turned into a content-based indexing device onto other knowledge resources. This affordance plays an important role in supporting the exploration of a scientific experiment and specifically for GED analysis [28]. A more in-depth analysis of affordances of FCA for the analysis and synthesis of Information Retrieval systems is [36].

This means that the data-driven lattices obtained with $\mathcal K$-Formal Concept Analysis can be used to support interaction with those ontologies by indexing them based in extents and intents. Besides, this also facilitates Gene Set Enrichment enabling the search of possible additional genes of GO terms present in a given bicluster and the evaluation of their hierarchy in the lattice.

We have enriched *WebGeneKFCA* with direct links to Gene Ontology (GO) and KEGG web services providing an interactive guide for navigating already available genetic information resources under the scope of the particular experiment being explored.

In particular, *WebGeneKFCA* provides a brief description for each probeset selected from a bicluster. This description has references to the NCBI gene database^8^ to ease the access to the latest online description of that gene. There are also references to each of the Gene Ontologies^9^ the gene belongs to, with one link to each ontology description. Finally *WebGeneKFCA* shows if the gene is known to belong to a pathway and provides a link to KEGG pathway database. ^10^

#### Statistical methods for hypothesis testing

Most EDA processes need to undergo a subsequent CDA step to empirically verify the discoveries. Prior to this costly process however, it is also convenient to calculate the probability for obtaining these gene clusters by looking at biological information known a priori, that is, computing the so-called *p-values* of the discovered biclusters with respect to well-known gene ontologies.

This idea was put on practice by [37] where different genes where classified in one of the different 199 functional categories from the *Martinsried Institute of Protein Sciences* (MIPS) yeast database and with this information the probability that each gene belongs to a cluster just by chance is calculated. In [38] the same work is done but with the a priori information gathered from the Gene Ontology (GO) database.

We follow the work of [39], developed in the context of *Gene Set Enrichment and Depletion* applications, a very important application favored by our setting, taking advantage of the fact that WebGeneKFCA provides interfacing facilities to GO (see section Interfacing with gene ontologies). The mathematical details have been included in Additional file 1.

## Results

### A methodology for the EDA of genomic expression data using concept lattices

#### A general view of the methodology

We propose the following stages for the interactive exploratory analysis of GED data with concept lattices.

1. Contextualization and data preparation
2. Exploration: concept lattice cardinality.
3. Exploration: gene and condition bi-clustering.
  ∙ Lattice-based under-expression analysis.
  ∙ Lattice-based over-expression analysis.
4. Exploration: Gene Set analysis.
  - Lattice-based functional enrichment with ontologies.
  - Lattice-based gene set enrichment analysis.

At each of these steps, the system provides input guidance and output visualization to assist and guide the user in hypothesis abduction.

Next we visit each of these stages clarifying them and providing a running example on real data.

#### Contextualization and data preparation

Contextualization refers to building a formal context or the data table for the analysis. The decisions typically affect whether to analyze in terms of genes or probesets and a detailing of what the conditions chosen for the study are, possibly with an explanation of what their purpose is.

Once the expression data have been gathered, they have to be rendered adequate for ulterior $\mathcal K$-FCA. To render data into a logarithmic cost amenable to $\overline {\mathbb {R}}_{\max,+}$ and $\overline {\mathbb {R}}_{\min,+}$ modeling, we suggest it should be normalized an log-compressed, in addition to any preprocessing to adjust for background noise, etc., [40].

**Running example: contextualization and data preparation** To illustrate our EDA process we have taken as a reference the *trisomy data* and the results presented in [41] where one chromosome 21 is silenced in pluripotent stem cells presenting trisomy 21. This extra chromosome is silenced by inserting a XIST gene found in the X chromosome which condenses one X chromosome in female mammals. In this experiment a modified version of the XIST gene was inserted into chromosome 21 and it was activated or deactivated by the presence of *Doxycycline*.

To build the context we downloaded from the National Center for Biotechnology Information [42] 27 CEL files from [41] (see Additional file 2). Only the probesets for chromosome 21 were selected so |*G*|=621 and the original expression data matrix has a dimension of 621×27.

Since there were 9×3 samples of different tissues, to average noise out the geometric mean of the expression level of each probeset was taken for each different tissue (3 samples of each), so that |*C*|=9 and we obtain a matrix *A* of 621×9 entries. The value of each probeset was later normalized by its overall geometric mean over the conditions and log-compressed

6 $$\begin{array}{*{20}l} r_{ij} = \log\frac{a_{ij}}{\sqrt[m]{\prod_{k=0}^{m} a_{ik}}} \end{array} $$

to obtain the context (*G*,*C*,*X*).

More details about this experiment can be found in Additional file 2 and it can be browsed on-line at https://webgenekfca.com/webgenekfca/datamatrices/7 for the data and https://webgenekfca.com/webgenekfca/kfcaresultses/9 for lattice exploration.

#### Exploration: choosing the range of thresholds for under- and over-expression

$\mathcal K$-FCA is a generic technique in $\mathcal K$ that, with the proper choice of thresholds and underlying cost algebras $\mathcal K$, allows us to explore gene over- and under-expression (see [27,*§* 2.2.2] for the details).

1. For each under-expression threshold *φ*, kFCA finds those biclusters maximal for inclusion of gene and condition sets that have a norm below the threshold
  7 $$\begin{array}{*{20}l} {\mathfrak{B}}^{\varphi}_{\max} (G, C, X) = \left\{ (a,b) \mid \| (a,b) \|_{\max} \leq \varphi\right\}. \end{array} $$
2. For each over-expression threshold *ϕ*, kFCA finds those biclusters minimal for inclusion of gene and condition sets that have a norm above the threshold *ϕ*,
  8 $$\begin{array}{*{20}l} {\mathfrak{B}}^{\phi}_{\min} (G, C, X) = \left\{ (a,b) \mid \| (a,b) \|_{\min} \geq \phi\right\}. \end{array} $$

Thus, for each matrix of GED, we need to explore in two directions to find both the under- and over-expressed genes in the conditions under scrutiny, but for the purpose of limiting the impact of under- and over-expression noise we want to avoid exploring values of the threshold close to 0.

Therefore, to analyze under-expression we explore in *φ*∈(−*∞*,0] and to analyze over-expression in *ϕ*∈[0,*∞*).

To guarantee the evolution of the exploration from threshold to threshold, we concentrate on those *φ* and *ϕ* that actually appear in the expression data, instead of exploring the whole ranges given. In such case, the product of the exploration are the sequences of lattices obtained with either threshold:

9 $$ \left\{{\mathfrak{B}}^{\varphi}_{\max} (G, C, X) \mid \varphi \in (-\infty, 0]\cap \{X_{ij}\}, i \leq m, j \leq n\right\}\,  $$

for under-expression and

10 $$ \left\{{\mathfrak{B}}^{\phi}_{\min} (G, C, X) \mid \phi \in [0,\infty)\cap \{X_{ij}\}, i \leq m, j \leq n\right\}\,  $$

for over-expression. Several other strategies could also be used to further decimate these ranges, like sampling the bins in a histogram analysis of *X*_*ij*_ values.

### The evolution of the number of concepts with thresholding

An important feature of the sequence of lattices produced in the exploration is the evolution of the *number of concepts or biclusters* along the *φ* and *ϕ* thresholds, since it provides an indication of the size and complexity of the lattice. For instance, in over-expression, if the absolute value of the threshold of existence is large, then large absolute values of the gene concentrations will be required for the concepts to exist, meaning that the lattice will only show the most salient relationships or biclusters. On the contrary, if the absolute values required for existence are low, many nodes of the lattice appear showing spurious relations due to the uncertainty during data collection or measurement noise.

Sudden changes in the slope reveal values of threshold where the lattice changes substantially and therefore will be values of interest to look into in the exploration process as we describe below.

**Fig. 2:**
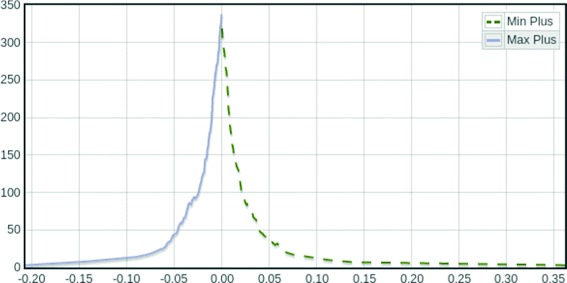
Evolution of the number of concepts [color on-line] Number of concepts vs. *φ* (*light blue*, continuous) to the left for *φ*<0.0, and number of concepts vs. *ϕ* (*drab green, dashed*) to the right for *ϕ*>0.0, for the context being explored

**Running example: visualizing the number of concepts** Figure 2 is a depiction of this evolution for the data described in the section Contextualization and data preparation.

Since *φ*∈(−*∞*,0] and *ϕ*∈[0,*∞*) the left part of the curve corresponds to under-expression analysis whilst the right one represents over-expression. The maximum number of concepts is attained at $\varphi = \phi = 0.0, |{\mathfrak {B}}^{0.0}_{\max } (G, C, X)| = |{\mathfrak {B}}^{0.0}_{\min } (G, C, X)| = 350$ well under the theoretical maximum at 2^|*C*|^=2^9^=512 : we take this to imply that the combinatorics of the probesets in this example is greatly reduced, supporting the hypothesis of heavy coregulation. In this case the interesting ranges to explore for under-expression and over-expression are, respectively, *φ*∈[−0.07,−0.02] and *ϕ*∈[0.02,0.08].

#### Exploring for under- and over-expression: visualization of bicluster hierarchy

The hierarchical organization of the bicluster lattices can be gleaned from the visualization scheme presented in Lattice visualization adaptation for GED. We say that the *higher* in the lattice a node appears, the more *abstract* the bicluster is, since the set of genes that comprise its extent appear in fewer conditions. Conversely, the *lower* the bicluster, the more *specific* it is since the set of genes included in its extent respond to a larger number of conditions and hence are better *profiled*.

When using the online tool, the gene appearance order for a given cluster as a function of *φ* is related to the confidence of that gene to belong to the given cluster. From there the reader can explore the lattices for different values of *φ* and *ϕ*.

##### Running example: gene under-expression analysis

**Fig. 3:**
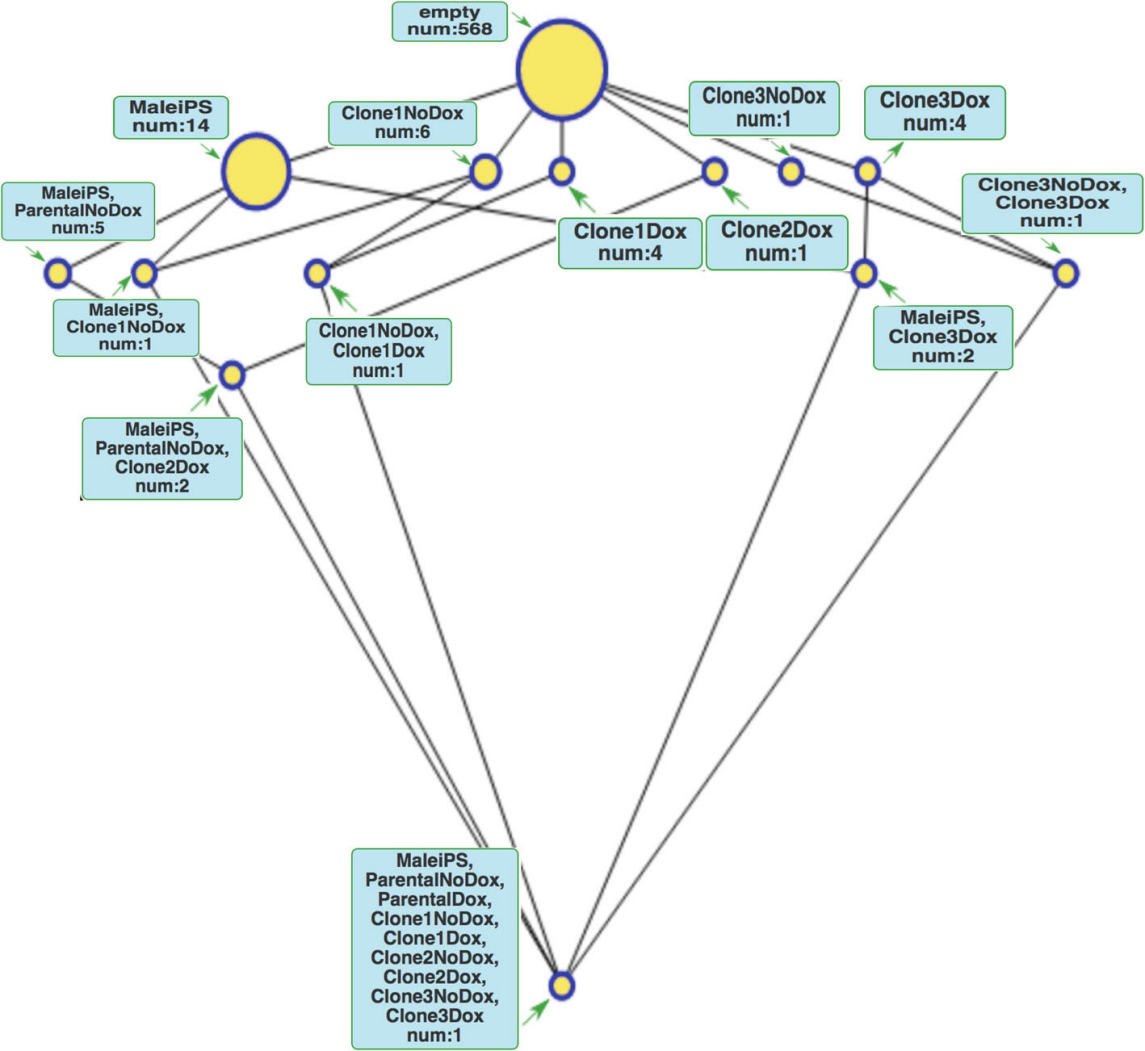
Gene under-expression lattice for *φ*=−0.150. This lattice can be browsed at https://webgenekfca.com/webgenekfca/kfcaresultses/9

If we select a very restrictive *φ*, i.e. if *φ*≪0 (see for example Fig. 3, *φ*=−0.150), only biclusters on the second row (those which are highly under-expressed in only one condition), a very few on the third (those highly under-expressed on two conditions) and a single one on the fourth (highly under-expressed on three conditions) appear^11^. That is, very *abstract* biclusters are present and the number of probesets that they contain is very low. Most of the probesets are at the *top* bicluster, meaning that we cannot assert that they are *under-expressed* with this very strict confidence level.

**Fig. 4:**
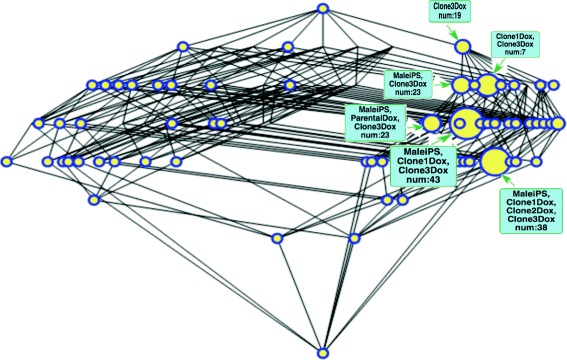
Gene under-expression lattice for *φ*=−0.010. This lattice can be browsed at https://webgenekfca.com/webgenekfca/kfcaresultses/9

As we relax the restrictions on *φ* more interesting groupings appear. In particular, around *φ*≈−0.010 (Fig. 4) big biclusters appear at the third, fourth and even fifth row allowing us to observe which sample combinations share the same under-expressed genes, notably situated to the right of the lattice where the condition *Clone3Dox* is placed. Specially relevant is the bicluster corresponding with *Male iPS* and all the *Clones* treated with *Doxycycline* (fifth row) that appears at this level but persists (i.e. is very *robust*) until *φ*=0.0. This way the interpretation that there are genes from the *Clones* treated with *Doxycycline* that have a similar level of expression as those found in disomatic cells is consistent with that given in the original paper [41] where only some genes from the trisomic cells are expected to decrease their expression when the third chromosome is silenced with the procedure there described.

Also worth mentioning is the biggest bicluster with 43 own probesets whose intent comprises Male iPS, and Clones 1 and 3 treated with Doxycycline. For some reason these two clones are responding slightly better than Clone 2 to the experiment, which is also consistent with the graph facilitated in [41, Fig. 4a]. Attending to the bicluster hierarchy, made evident in the lattice structure, we can say that this is a less *specific* bicluster than the one mentioned in the previous paragraph since these 43 probesets are the ones under-expressed in the three mentioned conditions but not for *Clone 2*.

Following the same line of reasoning, we can explore the third row where the two most significant biclusters are the ones for the conditions *MaleIPS* and *Clone3Dox* to the left and *Clone1Dox* and *Clone3Dox* to the right. These are parent nodes for the one described in the previous paragraph and ancestors of the one in the fith row mentioned earlier. Thus we can say that these are even less specific. This means that the probesets that we find there listed are under-expressed only in their respective pair of conditions but not in the rest.

**Fig. 5:**
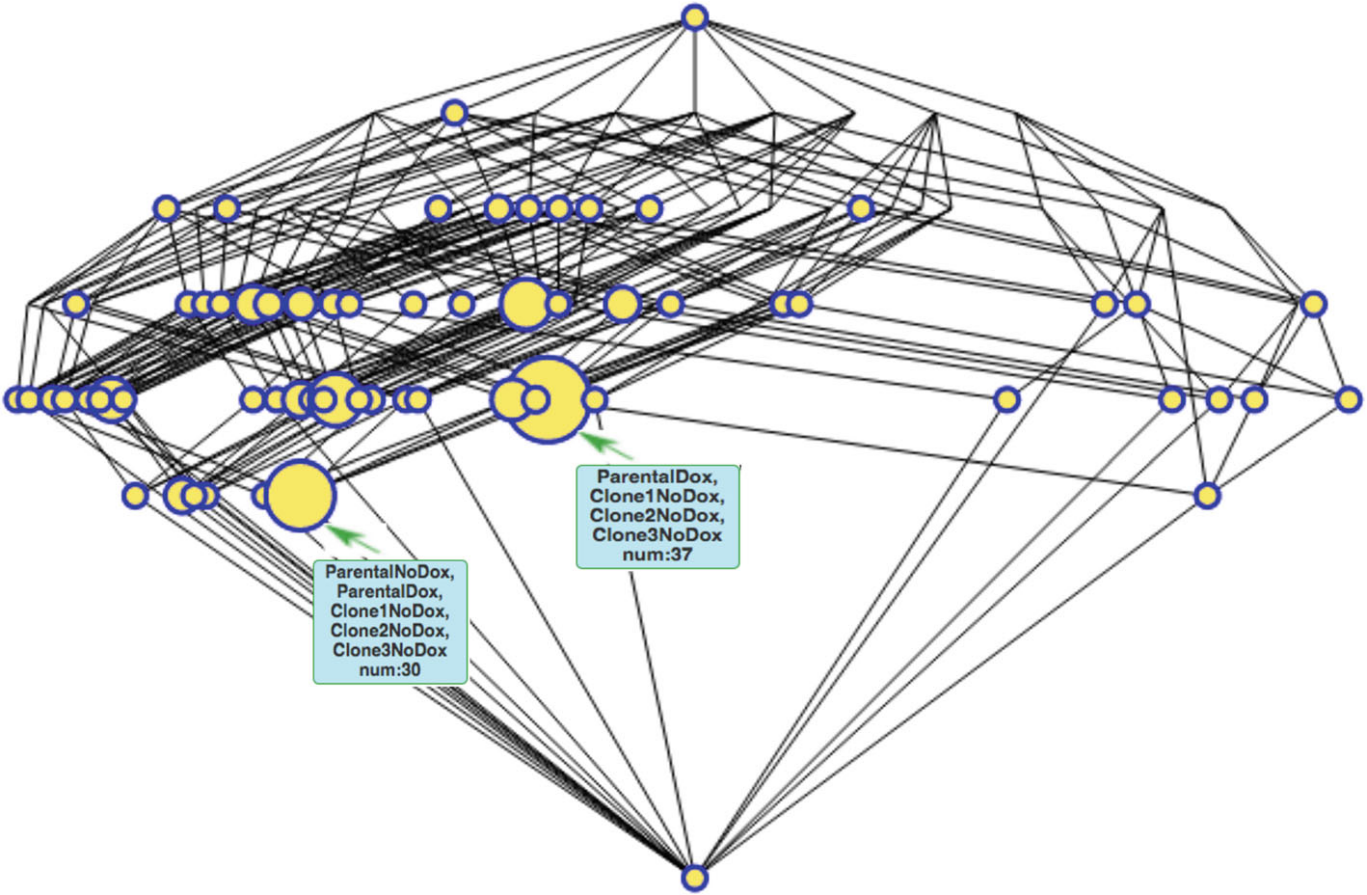
Gene under-expression lattice for *φ*=−0.005. This lattice can be browsed at https://webgenekfca.com/webgenekfca/kfcaresultses/9

But if we keep relaxing the restrictions approaching the origin at *φ*≈−0.005 (Fig. 5) big biclusters appear yet in lower positions of the lattice (fith and sixth rows in the example) and therefore are more *specific*. However, let us remind that for these very specific biclusters to appear, extremely low variations in the concentrations are admitted as significant and the results are prone to measurement errors. The explorer should be cautious when analysing these biclusters. See, for example, that the big bicluster in the sixth row shows the genes that are under-expressed (minimally) to both *Parental* (with and without *Doxicicline* treatment) and all the non-treated clones.

##### Running example: gene over-expression analysis

In the analysis of over-expression the exploration order is reversed. As can be noted from Fig. 2, a larger number of concepts, presumably including too specific biclusters with low reliability, appear at lower values of *ϕ* due to the relaxed restrictions on the concentrations and more robust biclusters can be observed with larger values of *ϕ*.

**Fig. 6:**
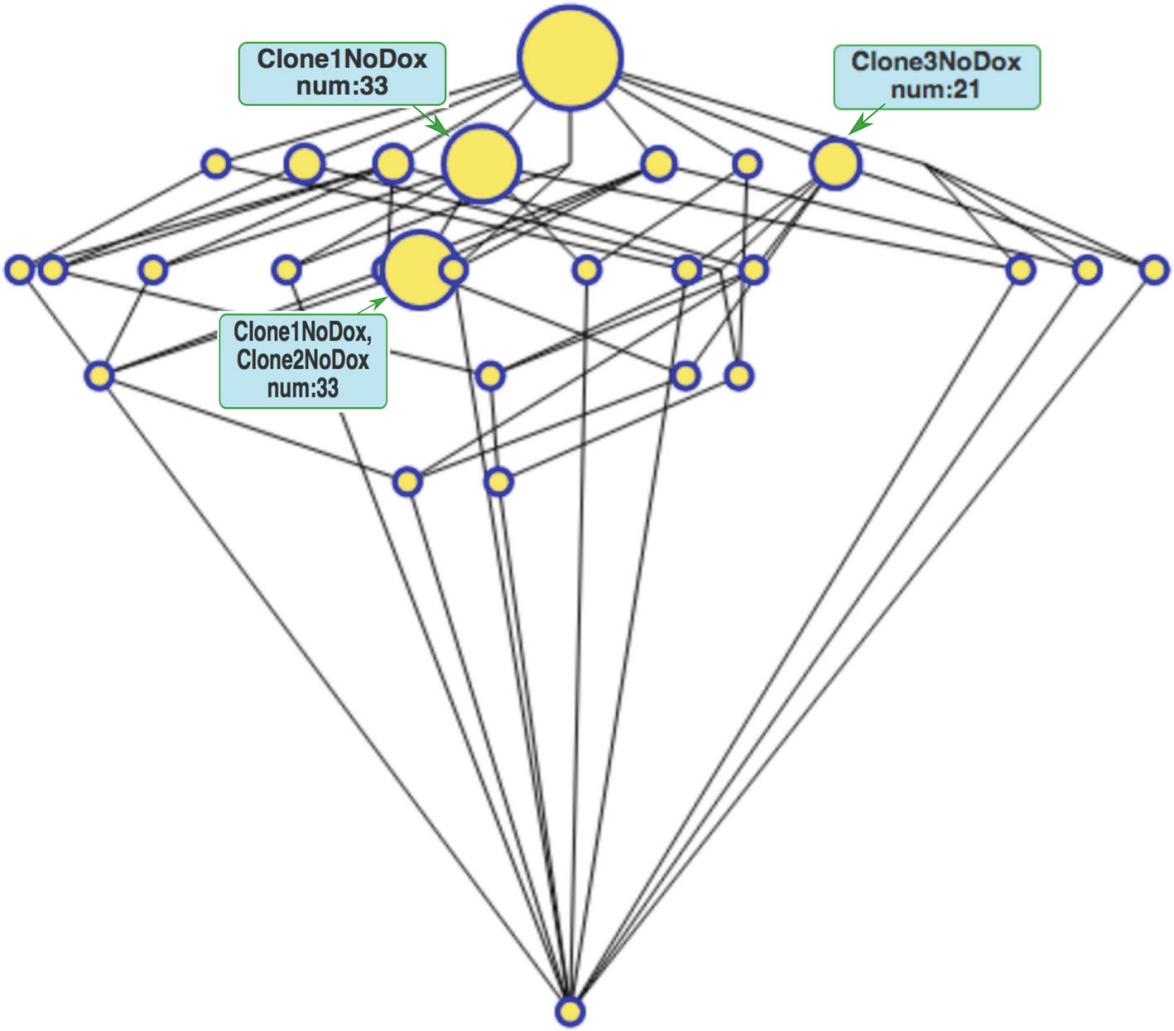
Gene over-expression lattice for *ϕ*=0.05. This lattice can be browsed at https://webgenekfca.com/webgenekfca/kfcaresultses/9

As an example for *ϕ*=0.05 (Fig. 6) there is one big cluster in the third row for the non-treated Clones 1 and 2 and two others in the second row again for the non-treated Clone 1 and non-treated Clone 3 showing that, in this case, over-expression rather affects the non-treated samples.

#### Exploration: gene set analysis

Next, as part of the lattice exploration, the user can evaluate the lists of probesets of each node making use of the interfacing facilities with external ontologies and also obtain their *p-values* as indicated in Interfacing with gene ontologies.

##### Running example: interfacing with GO

A print-out of a sample information that can be obtained by interfacing with GO through the lattices is included in Additional file 3. Please, note that the links in *WebGeneKFCA* are active and lead to the ontologies’ on-line databases. This particular sample has been obtained by digging into the main cluster of the lattice of Fig. 4 and selecting the probeset 11742211_x_at.

##### Running example: gene enrichment

This principle can be applied to the clusters identified in the previous section. For example, Table 1 shows the ten most reliable – the ones with the lowest *p-values* – gene ontology terms from two different clusters. In this case the genes have been replaced by the microarray probesets. The full list can be obtained on-line at https://webgenekfca.com/webgenekfca/kfcaresultses/9 as a CSV file and also as Additional file 4. A print-out is also included in Additional file 5.

**Table 1:** Ten most significant GO terms including their *p*-values computed as described in Additional file 1 – Statistical methods for hypothesis testing for the bicluster with intent *MalePS*, *Parental NoDox*, *Clone 1 NoDox* and *Clone 3 NoDox* from Fig. 4

GO term	Description	Ont.	*p*-value	probesets
GO:0005905	Coated pit	CC	1.11E-16	11742211_x_at, 11742215_s_at
				11746866_a_at, 11752199_a_at
				11752200_x_at, 11738008_s_at
				11741216_x_at, 11742210_a_at
				11751350_a_at, 11750384_a_at
				11752481_a_at
GO:0016358	Dendrite development	BP	1.11E-16	11742211_x_at, 11742215_s_at
				11746866_a_at, 11752199_a_at
				11752200_x_at, 11738008_s_at
				11741216_x_at, 11742210_a_at
				11751350_a_at, 11750384_a_at
				11752481_a_at
GO:0008344	Adult locomotory behavior	BP	1.11E-16	11742211_x_at, 11746866_a_at
				11752199_a_at, 11757287_x_at
				11742210_a_at, 11751350_a_at
				11750384_a_at, 11728320_a_at
				11742215_s_at, 11752200_x_at
				11738008_s_at, 11741216_x_at
				11752481_a_at
GO:0035253	Ciliary rootlet	CC	1.11E-16	11742211_x_at, 11742215_s_at
				11746866_a_at, 11752199_a_at
				11752200_x_at, 11738008_s_at
				11741216_x_at, 11742210_a_at
				11751350_a_at, 11750384_a_at
				11752481_a_at
GO:0006378	mRNA polyadenylation	BP	1.11E-16	11742211_x_at, 11742215_s_at
				11746866_a_at, 11752199_a_at
				11752200_x_at, 11738008_s_at
				11741216_x_at, 11742210_a_at
				11751350_a_at, 11750384_a_at
				11752481_a_at
GO:0048669	Collateral sprouting in absence of injury	BP	1.11E-16	11742211_x_at, 11742215_s_at
				11746866_a_at, 11752199_a_at
				11752200_x_at, 11738008_s_at
				11741216_x_at, 11742210_a_at
				11751350_a_at, 11750384_a_at
				11752481_a_at
GO:0030900	Forebrain development	BP	1.11E-16	11742211_x_at, 11742215_s_at
				11746866_a_at, 11752199_a_at
				11752200_x_at, 11738008_s_at
				11741216_x_at, 11742210_a_at
				11751350_a_at, 11750384_a_at
				11752481_a_at
GO:0019717	Synaptosome	CC	1.11E-16	11742211_x_at, 11746866_a_at
				11752199_a_at, 11722552_x_at
				11722551_s_at, 11742210_a_at
				11751350_a_at, 11750384_a_at
				11742215_s_at, 11752200_x_at
				11738008_s_at, 11741216_x_at
				11736141_a_at
				11752481_a_at
GO:0004867	Serine-type endopeptidase inhibitor activity	MF	1.11E-16	11742211_x_at, 11742215_s_at
				11746866_a_at, 11752199_a_at
				11752200_x_at, 11738008_s_at
				11741216_x_at, 11742210_a_at
				11751350_a_at, 11750384_a_at
				11752481_a_at
GO:0043197	Dendritic spine	CC	2.22E-16	11742211_x_at, 11742215_s_at
				11746866_a_at, 11752199_a_at
				11752200_x_at, 11738008_s_at
				11741216_x_at, 11742210_a_at
				11751350_a_at, 11750384_a_at
				11752481_a_at

The full list can be obtained on-line at https://webgenekfca.com/webgenekfca/kfcaresultses/9 as a CSV file or as Additional file 4 – CSV le of GO terms’ information for gene enrichment from WebGeneKFCA. A print-out is also included in Additional file 5 – Print-out of GO terms’ information for gene enrichment from WebGeneKFCA

## Discussion

The aim of this paper is not merely to introduce a new biclustering algorithm for GED analysis but instead to offer a set of analysis and visualization tools that revolve around $\mathcal {K}$-Formal Concept Analysis. These are cast into the framework of Exploratory Data Analysis (EDA) to support full human interaction during the step of hypothesis abduction. Tukey was the figure who advocated the use of exploratory, discovery-driven methods in the Statistics community. He coined the expression “Exploratory Data Analysis” and supported a complementary curriculum for Statisticians balancing EDA against Statistical Hypothesis Testing (that he called Confirmatory Data Analysis) [10]. At present, EDA is standard practice of good statistics, it is taught at basic statistics courses in academia [43], and supported by widely-used data processing software [44, 45].

The reader should be aware that since this methodology cannot be used as a tool for Confirmatory or Predictive Data Analysis by itself, an empirical verification of the hypothesis gained in the exploration should be carried out as a subsequent step. We believe, nevertheless, that the framework we promote is specially suited to support scientific knowledge discovery in GED [28] given the usual lack of ground-truth in this type of data. This leads us to the enduring dilemma of obtaining quality indicators in an EDA framework.

Despite not being a Confirmatory Data Analysis tool, our framework profits from some affordances of FCA-related tools to define quality indicators. On the one hand, we introduced the notion of *persistence* or *robustness* of a bi-cluster based on the range of *φ* for which it exists—thus providing an idea of a concept robustness—, and the degree of confidence on the empirical measures that define the bi-clusters [29].

On the other hand, the resulting conceptual lattice can be used to index external databases, such as Gene Ontology (GO), thus offering a new way of accessing other available resources. According to the theoretical background of Gene Set Enrichment, this enables the calculation of the *p*-values of bi-clusters as confidence measures based on the terms in those resources. In this sense, the sequence of lattices for a particular experiment allows the researcher to observe the evolution of a gene throughout the different biclusters it appears in, thus vertebrating the researcher vision of that given resource. This may be used to confer relevance to bi-clusters, observing which genes are included and what their persistence is, to infer, for example, hypotheses on their function.

Another key aspect of an EDA framework is its data visualization capabilities. We profit from two important FCA affordances in this regard: namely, that the formal quality of FCA makes it suitable for domain-independent data analysis, and that visualization and manipulation of data in table format and hierarchical format are mutually warranted—stemming from the duality of contexts and lattices. Furthermore, since we extend FCA to support non boolean data tables by using $\mathcal K$-FCA, our visualization of GED takes the form of a sequence of lattices.

An important consideration in the exploration of this sequence of lattices is the choice of the particular values of *φ* and *ϕ* of interest. Hints for the discovery of relevant positions based on the evolution of the number of biclusters (or concepts) are presented in The evolution of the number of concepts with thresholding but it is worth noting that values of *φ* and *ϕ* close to 0 can be error-prone in noisy samples. As we have mentioned before, higher absolute values of these thresholds will show genes more intensely over- or under-expressed, perhaps at the expense of missing some other relations. A current limitation of this representation is the small number of samples or conditions that can be simultaneously visualized, a disadvantage that can be partially ameliorated by a pre-processing stage providing manually arranged groupings of the conditions. But this ought to be revisited in the future.

## Conclusions

Within the framework of EDA, we have introduced a set of interactive analysis tools based on $\mathcal K$-FCA that include a new bi-clustering algorithm for GED analysis and visualization capabilities to support exploration of GED backed by two quality indicators: our own defined *persistence* of a bi-cluster and the *p-values* computed within the background of Gene Set Enrichment statistical confidence measures facilitated by the indexing capabilities of $\mathcal {K}$-FCA for external databases, such as Gene Ontology.

In contrast with the currently dominant paradigm of Confirmatory or Predictive Data Analysis, with important difficulties for its application to GED intrinsic to the problem definition and mainly due to the lack of ground-truth data, we belief that framing GED analysis in an EDA setting (possibly complemented with ulterior empirical verification of the findings) is a principled and relevant change of paradigm in GED analysis that eases the understanding of the process of scientific discovery. We have illustrated the capabilities of our $\mathcal {K}$-FCA-based EDA framework with the analysis of a real data set confirming previously published findings on that data.

The exploration facilities that it offers stem from two main abilities of $\mathcal {K}$-FCA: first, as an FCA-derived methodology, it can be interpreted as a non-partitional bi-clustering method that can be visualized as a sequence of lattices, evidencing the hierarchical structure of the biclusters thus obtained and already facilitating browsing into its structure and second, by using either max-plus or min-plus as the underlying semirings, we obtain interpretations for gene *under- and over-expression* respectively with a free parameter, the *threshold of expression*, we have used to define an extra flavor of exploratory analysis: changing this value, the level of detail at which the context is being explored varies, entailing differences in the lattice. Indeed, Fig. 2 is an exploratory plot of the consequences of varying such parameter in the particular case of analyzing gene expression.

By the previous procedure, the GED analysis problem gets transformed into the exploration of a *sequence of lattices* enabling the visualization of the hierarchical structure of the biclusters with a certain degree of granularity. A crucial advantage of our graphical representation of this sequence is the guarantee that all the biclusters with the same set of conditions are always plotted in the same spatial coordinates, therefore facilitating their interpretation.

The graphical interface, *WebGeneKFCA*, as an interactive tool for analysis and decision, allows the user to navigate along this parameter to observe rougher or finer biclusters. The algorithm, available as a web service, allows the researcher to analyze gene expression data with no previous knowledge of the experiment conditions and also interface with external gene ontologies.

## Endnotes

^1^http://www.ncbi.nlm.nih.gov/gene

^2^http://amigo.geneontology.org/amigo/

^3^http://www.genome.jp/kegg/pathway.html

^4^Provided $\mathbb R$ has the structure of a completed idempotent semifield ${\mathcal K}$, a technicality to induce the lattice structure.

^5^To ensure that every bicluster with the same set of conditions appears at the same position throughout the whole sequence of lattices, each lattice is drawn *over* the silhouette of the Concept Lattice of a (virtual) contranominal scale involving all possible attributes, $\mathbb {N^{c}_{M}} = \underline {\mathfrak {B}}(M,M,\neq)$. The rationale for this is explained in [29].

^6^ In the on-line tool *WebGeneKFCA* only the label for the node selected with the mouse is shown to avoid cluttering the diagram.

^7^ To complete the list of all the genes under-expressed for that condition we need to add those from that bicluster downwards in the lattice: from row 3, 5 genes from the node below to the left that corresponds to the conditions *MaleIPS* and *ParentalNoDox*, 1 gene from the node right below with conditions *MaleIPS* and *Clone1NoDox* and 2 genes from the node below to the left of the lattice with the conditions *MaleIPS* and *Clone2Dox* and from row 4,2 more genes corresponding to the node with *MalIPS*, *ParentalNoDox* and *Clone2Dox*.

^8^http://www.ncbi.nlm.nih.gov/gene

^9^http://amigo.geneontology.org/amigo/

^10^http://www.genome.jp/kegg/pathway.html

^11^Recall from section Lattice visualization adaptation for GED that the top node is in row 1.

## Additional files

Additional file 1 Statistical methods for hypothesis testing. Description of the statistical methods for hypothesis testing employed in the paper and implemented in the on-line service *WebGeneKFCA*. (PDF 141 kb)

Additional file 2 Data procurement and normalization of the running example. Details about the procurement and normalization of the data employed in the running example employed throughout the paper. (PDF 171 kb)

Additional file 3 Print-out of the genomic information interfaced by *WebGeneKFCA*. A print-out of a sample information that can be obtained by interfacing with GO through the lattices of the running example described in http://www.biomedcentral.com/content/supplementary/10.1186/s12859-016-1234-z-S1.pdfAdditional file 1. Please, note that the links in *WebGeneKFCA* are active and lead to the ontologies’ on-line databases. This particular sample has been obtained by digging into the main cluster of the lattice of Fig. 4 and selecting the probeset 11742211_x_at. It can also be obtained at https://webgenekfca.com/webgenekfca/kfcaresultses/9. (PDF 471 kb)

Additional file 4 CSV file of GO terms’ information for gene enrichment from *WebGeneKFCA*. A sample print-out from *WebGeneKFCA* with the information about the GO terms and their *p*-values computed as described in http://www.biomedcentral.com/content/supplementary/10.1186/s12859-016-1234-z-S2.pdfAdditional file 2 for the bicluster with intent *MalePS*, *Parental NoDox*, *Clone 1 NoDox* and *Clone 3 NoDox* from Fig. 4. It can also be obtained on-line at https://webgenekfca.com/webgenekfca/kfcaresultses/9. (CSV 24 kb)

Additional file 5 Print-out of GO terms’ information for gene enrichment from *WebGeneKFCA* A sample print-out from *WebGeneKFCA* with the information about the GO terms and their *p*-values computed as described in http://www.biomedcentral.com/content/supplementary/10.1186/s12859-016-1234-z-S2.pdfAdditional file 2 for the bicluster with intent *MalePS*, *Parental NoDox*, *Clone 1 NoDox* and *Clone 3 NoDox* from Fig. 4. It can also be obtained on-line at https://webgenekfca.com/webgenekfca/kfcaresultses/9 as a CSV file. (PDF 358 kb)
