# Supplementary material for: Interactive knowledge discovery and data mining on genomic expression data with numeric formal concept analysis
Source: BMC Bioinformatics. 2016 Sep 15;17:374. doi: 10.1186/s12859-016-1234-z (PMC5024470; doi:10.1186/s12859-016-1234-z)
Supplement: Additional file 1 — Statistical methods for hypothesis testing. Description of the statistical methods for hypothesis testing employed in the paper and implemented in the on-line service WebGeneKFCA. (PDF 141 kb) [file 12859_2016_1234_MOESM1_ESM.pdf]

SUPPLEMENTARY MATERIALS: STATISTICAL HYPOTHESIS TESTING

Interactive Knowledge Discovery and Data Mining on Genomic Expression Data with Numeric Formal Concept Analysis

Jose M González-Calabozo[1], Francisco J Valverde-Albacete[1] and Carmen Peláez-Moreno[1*]

*Correspondence: carmen@tsc.uc3m.es
[2]Department of Signal Theory and Communications, University Carlos III Madrid, Avda. Universidad, 30, Leganés (Madrid), Spain
Full list of author information is available at the end of the article

Supplementary Materials: Statistical methods for hypothesis testing

We follow the work of [1], developed in the context of *Gene Set Enrichment and Depletion* applications, a very important application favoured by our setting, taking advantage of the fact that WebGeneKFCA provides an interfacing facilities to GO: recall there are m genes is the total genome population in an experiment and that given a bicluster contains g genes and a GO category with f genes a contingency matrix like the one in Table 1 can be obtained. Our work is to find out whether the bicluster induced by the lattices constitutes an enrichment, a depletion or neither of them.

Table 1 Gene counts depending on the GO functional category.

	$\in$ GOTerm	$\notin$ GOTerm	Total
**bicluster**	k	$g-k$	g
**rest**	$f-k$	$m-g+k-f$	$m-g$
**Total**	f	$m-f$	m

Let H_0, be the null hypothesis be that the genes of a bicluster have been picked at random from the total gene population. Using the hyper-geometric formulation, the random variable K whose realization is the observed value k is distributed according to $\sim$ hyper(m, g, f)

$$P(k \mid H_0) = \frac{\binom{f}{k}\binom{m-f}{g-k}}{\binom{m}{g}} \tag{1}$$

Let p_1 be the probability that the genes in the bicluster of interest belong to the GO category and p_2 the probability that those not in the bicluster belong to that category. Then, $p_1 = p_2$ is the null hypothesis and the alternative hypotheses are:

- For the condition of *enrichment* (E) "if $H_a = E$, then $p_1 > p_2$" with $p_1 = P(K \geq k \mid H_0)$.
- For the condition of *depletion* (D) "if $H_a = D$, then $p_1 < p_2$" with $p_1 = P(K \leq k \mid H_0)$.

For a required significance level—or probability for type I errors—α we "reject H_0 if $p_1 \leq \alpha$" and we infer that the GO term in question is enriched or depleted, as adequate.

From (1), either one-tailed or two-tailed statistics can be used [1]. But since we want to treat enrichment and depletion differently, we use one-tailed statistics. Note that in [2, 3, 4] a one-tailed test statistic defined as the probability to find at least k genes from one functional category, assuming as null hypothesis that the cluster does not classify for functional category, that is, testing for enrichment:

$$P(K \geq k \mid H_0) = 1 - P(K < k \mid H_0) \tag{2}$$

Author details

[1]Department of Signal Theory and Communications, University Carlos III Madrid, Avda. Universidad, 30, Leganés (Madrid), Spain. [2]Department of Signal Theory and Communications, University Carlos III Madrid, Avda. Universidad, 30, Leganés (Madrid), Spain.

References

1. Rivals, I., Personnaz, L., Taing, L., Potier, M.-C.: Enrichment or depletion of a GO category within a class of genes: which test? Bioinformatics **23**(4), 401–7 (2007). doi:10.1093/bioinformatics/btl633
2. Jiang, D., Tang, C., Zhang, A.: Cluster analysis for gene expression data: a survey. IEEE Transactions on Knowledge and Data Engineering **16**(11), 1370–1386 (2004). doi:10.1109/TKDE.2004.68
3. Tavazoie, S., Hughes, J.D., Campbell, M.J., Cho, R.J., Church, G.M.: Systematic determination of genetic network architecture. Nat. Genet. **22**(3), 281–5 (1999). doi:10.1038/10343
4. Berriz, G.F., King, O.D., Bryant, B., Sander, C., Roth, F.P.: Characterizing gene sets with FuncAssociate. Bioinformatics **19**(18), 2502–2504 (2003). doi:10.1093/bioinformatics/btg363
