# Supplementary material for: Interactive knowledge discovery and data mining on genomic expression data with numeric formal concept analysis
Source: BMC Bioinformatics. 2016 Sep 15;17:374. doi: 10.1186/s12859-016-1234-z (PMC5024470; doi:10.1186/s12859-016-1234-z)
Supplement: Additional file 2 — Data procurement and normalization of the running example. Details about the procurement and normalization of the data employed in the running example employed throughout the paper. (PDF 171 kb) [file 12859_2016_1234_MOESM2_ESM.pdf]

SUPPLEMENTARY MATERIALS: DATA PROCUREMENT AND NORMALIZATION

Interactive Knowledge Discovery and Data Mining on Genomic Expression Data with Numeric Formal Concept Analysis

Jose M González-Calabozo[1], Francisco J Valverde-Albacete[1] and Carmen Peláez-Moreno[1*]

*Correspondence:
carmen@tsc.uc3m.es
[2]Department of Signal Theory and Communications, University Carlos III Madrid, Avda. Universidad, 30, Leganés (Madrid), Spain
Full list of author information is available at the end of the article

Supplementary Materials: Data procurement and normalization

To illustrate the process of exploration with *WebGeneKFCA*, we have presented a running example of analysis intended as a guide through the interactive process that can be followed to discover the relationships among genes belonging to different human samples. The dataset supporting the conclusions of this article is available in the Gene Expression Omnibus (GEO) repository http://www.ncbi.nlm.nih.gov/geo/query/acc.cgi?acc=GSE47014. The webgenekfca tool employed for the analysis is available at https://webgenekfca.com/webgenekfca/

The analyzed tissues and the reference code for each experiment are:

- **Male iPS**: induced pluripotent stem cells from a disomic male used as a control group (three samples).
- **Parental no Dox**: induced pluripotent stem cells from cells with trisomy 21 used as a control group from cells without treatment (three samples).
- **Parental Dox**: induced pluripotent stem cells from cells with trisomy 21 and treated with *Doxycycline*. These samples are used as control to detect any possible effect of *Doxycycline* in the trisomic control cells (three samples).
- **Clone N no Dox**: induced pluripotent stem cells from cells with trisomy 21 with inserted XIST gene. Here, this gene is not expressed because the samples are not under a *Doxycycline* treatment (three independent subclones, each of them with three samples).
- **Clone N Dox**: induced pluripotent stem cells from cells with trisomy 21 with inserted XIST gene and treated with *Doxycycline* which makes the XIST gene expressed producing a condensation of one of the chromosomes 21 (three independent subclones, each of them with tree samples).

A new experiment in *WebGeneKFCA* was created by uploading the 27 gene expression Affymetrix CEL files. RMA (Robust Multichip Average) summarization was performed using APT (Affymetrix Power Tools) [1] resulting on a 49395×27 matrix containing an expression of a single probeset per row. The correspondence between genes and probesets [2] is given by the Affymetrix Annotation file. From these 49395 probesets only the 610 corresponding to the genes from chromosome 21 were retained.

Then, with the purpose of removing noise, samples from similar tissues were grouped and their value was substituted by the arithmetic mean of each of them. Thus the final matrix has $m = 610$ rows by $n = 9$ columns which will be labeled

as: *Male iPS*, *Parental no Dox*, *Parental Dox*, *Clone 1 no Dox*, *Clone 1 Dox*, *Clone 2 no Dox*, *Clone 2 Dox*, *Clone 3 no Dox* and *Clone 3 Dox*.

Prior to start the $\mathcal{K}$-Formal Concept Analysis exploration we need to perform some preprocessing to allow the comparison of the expression of different probesets. Since the different probesets have different ranges of expression, a normalization is necessary. Several alternatives are implemented in *WebGeneKFCA* (see [3]) and here we have chosen to apply the natural logarithm to each probeset i for the condition j divided by the geometric mean of the gene expression over all considered conditions:

$$r'_{ij} = \log \frac{r_{ij}}{\bar{r}} \tag{1}$$

where

$$\bar{r} = \frac{1}{p} \prod_{k=0}^{p-1} r_{ik} \tag{2}$$

Author details
[1]Department of Signal Theory and Communications, University Carlos III Madrid, Avda. Universidad, 30, Leganés (Madrid), Spain. [2]Department of Signal Theory and Communications, University Carlos III Madrid, Avda. Universidad, 30, Leganés (Madrid), Spain.

References

1. Affymetrix: Affymetrix Power Tools. http://www.affymetrix.com/partners_programs/programs/developer/tools/powertools.affx (2013)
2. Affymetrix: What is a probeset? http://www.affymetrix.com/support/help/faqs/mouse_430/faq_8.jsp (2013)
3. González-Calabozo, J.M., Peláez-Moreno, C., Valverde-Albacete, F.J.: Gene expression array exploration using K-Formal Concept Analysis. In: Valtchev, P., Jäschke, R. (eds.) Formal Concept Analysis. Lecture Notes in Computer Science, vol. 6628, pp. 119–134. Springer, Heidelberg (2011)
