# Supplementary material for: Interactive knowledge discovery and data mining on genomic expression data with numeric formal concept analysis
Source: BMC Bioinformatics. 2016 Sep 15;17:374. doi: 10.1186/s12859-016-1234-z (PMC5024470; doi:10.1186/s12859-016-1234-z)
Supplement: Additional file 3 — Print-out of the genomic information interfaced by WebGeneKFCA. A print-out of a sample information that can be obtained by interfacing with GO through the lattices of the running example described in http://www.biomedcentral.com/content/supplementary/10.1186/s12859-016-1234-z-S1.pdfAdditional file 1. Please, note that the links in WebGeneKFCA are active and lead to the ontologies’ on-line databases. This particular sample has been obtained by digging into the main cluster of the lattice of Fig. 4 and selecting the probeset 11742211_x_at. It can also be obtained at https://webgenekfca.com/webgenekfca/kfcaresultses/9. (PDF 471 kb) [file 12859_2016_1234_MOESM3_ESM.pdf]

This view shows Gene Ontology information about the selected cluster.
The following links provide with three different CSV files where the rows are linked to a probeset and the columns are the gene ontologies each probeset belongs. A 1 means that the probeset belongs to that particular ontology. Each file will provide information about *Biological Process*, *Molecular Function* or *Cellular Component*.

Download BP   Download MF   Download CC

This part shows a list of Gene Ontology terms sortered by their p-value. This p-value shows the probability, measured from 0 to 1, that the given Gene Ontology term has appeared in the cluster by chance. This can be used as a meassure of how reliable this cluster is, thus lower values are better.
The following table shows the most probable GO terms sorted by their probability of random appearance. The columns are the following:

- *GO term*:Gene ontology term accession name.
- *Name*: GO name.
- *Ratio cluster/total*: Number of times this GO term appears in the cluster compared with the times that appears in the microarray.
- *p-value*: Probability of random appearance.

Cluster size: 43 probesets
Cluster attributes: MaleiPS, Clone1Dox, Clone3Dox
Number of probesets in microarray: 610

Download

GO term	Description	Ontology	Ratio cluster/total	p-value	probeset
GO:0051124	synaptic growth at neuromuscular junction	BP	11/14	0.0000	11742211_x_at
GO:0031594	neuromuscular junction	CC	11/106	0.0000	11742211_x_at
GO:0051402	neuron apoptosis	BP	11/90	0.0000	11742211_x_at
GO:0016199	axon midline choice point recognition	BP	11/24	0.0000	11742211_x_at
GO:0033130	acetylcholine receptor binding	MF	11/24	0.0000	11742211_x_at
GO:0030414	peptidase inhibitor activity	MF	13/262	0.0000	11742211_x_at
GO:0043198	dendritic shaft	CC	11/89	0.0000	11742211_x_at
GO:0045931	positive regulation of mitotic cell cycle	BP	11/87	0.0000	11742211_x_at
GO:0007626	locomotory behavior	BP	11/198	0.0000	11742211_x_at
GO:0031175	neuron projection development	BP	11/197	0.0000	11742211_x_at
GO:0040014	regulation of multicellular organism growth	BP	11/89	0.0000	11742211_x_at
GO:0016504	peptidase activator activity	MF	11/34	0.0000	11742211_x_at
GO:0051563	smooth endoplasmic reticulum calcium ion homeostasis	BP	11/17	0.0000	11742211_x_at
GO:0035235	ionotropic glutamate receptor signaling pathway	BP	11/41	0.0000	11742211_x_at
GO:0007219	Notch signaling pathway	BP	11/196	0.0000	11742211_x_at
GO:0007617	mating behavior	BP	11/25	0.0000	11742211_x_at
GO:0050885	neuromuscular process controlling balance	BP	11/104	0.0000	11742211_x_at
GO:0016322	neuron remodeling	BP	11/28	0.0000	11742211_x_at
GO:0007176	regulation of epidermal growth factor receptor activity	BP	11/30	0.0000	11742211_x_at
GO:0008542	visual learning	BP	11/98	0.0000	11742211_x_at
GO:0045177	apical part of cell	CC	11/192	0.0000	11742211_x_at
GO:0050803	regulation of synapse structure and activity	BP	11/24	0.0000	11742211_x_at
GO:0001967	suckling behavior	BP	11/24	0.0000	11742211_x_at
GO:0000085	G2 phase of mitotic cell cycle	BP	11/25	0.0000	11742211_x_at
GO:0051233	spindle midzone	CC	11/28	0.0000	11742211_x_at
GO:0008088	axon cargo transport	BP	11/25	0.0000	11742211_x_at
GO:0005905	coated pit	CC	11/188	1.1102e-16	11742211_x_at
GO:0016358	dendrite development	BP	11/99	1.1102e-16	11742211_x_at
GO:0008344	adult locomotory behavior	BP	13/133	1.1102e-16	11742211_x_at
GO:0035253	ciliary rootlet	CC	11/46	1.1102e-16	11742211_x_at
GO:0006378	mRNA polyadenylation	BP	11/48	1.1102e-16	11742211_x_at
GO:0048669	collateral sprouting in absence of injury	BP	11/13	1.1102e-16	11742211_x_at
GO:0030900	forebrain development	BP	11/209	1.1102e-16	11742211_x_at
GO:0019717	synaptosome	CC	14/368	1.1102e-16	11742211_x_at
GO:0004867	serine-type endopeptidase inhibitor activity	MF	11/207	1.1102e-16	11742211_x_at
GO:0043197	dendritic spine	CC	11/227	2.2204e-16	11742211_x_at
GO:0006897	endocytosis	BP	14/452	2.2204e-16	11742211_x_at
GO:0006878	cellular copper ion homeostasis	BP	11/31	2.2204e-16	11742211_x_at
GO:0030198	extracellular matrix organization	BP	11/255	3.3307e-16	11742211_x_at
GO:0006417	regulation of translation	BP	11/284	8.8818e-16	11742211_x_at
GO:0007409	axonogenesis	BP	11/297	1.4433e-15	11742211_x_at
GO:0043005	neuron projection	CC	11/315	2.6645e-15	11742211_x_at
GO:0008201	heparin binding	MF	11/333	5.1070e-15	11742211_x_at
GO:0045202	synapse	CC	14/916	2.4869e-14	11742211_x_at
GO:0008219	cell death	BP	11/466	2.0628e-13	11742211_x_at
GO:0030424	axon	CC	11/504	4.8239e-13	11742211_x_at
GO:0006917	induction of apoptosis	BP	11/628	5.1440e-12	11742211_x_at
GO:0005102	receptor binding	MF	11/671	1.0447e-11	11742211_x_at
GO:0009986	cell surface	CC	11/883	1.9226e-10	11742211_x_at
GO:0006915	apoptosis	BP	14/1815	2.2554e-10	11742211_x_at
GO:0031410	cytoplasmic vesicle	CC	11/925	3.1342e-10	11742211_x_at
GO:0048471	perinuclear region of cytoplasm	CC	11/1134	2.6264e-9	11742211_x_at
GO:0042802	identical protein binding	MF	11/1172	3.6943e-9	11742211_x_at
GO:0045944	positive regulation of transcription from RNA polymerase II promoter	BP	11/1203	4.8377e-9	11742211_x_at
GO:0008233	peptidase activity	MF	11/1366	1.7832e-8	11742211_x_at
GO:0007155	cell adhesion	BP	12/1744	2.0168e-8	11742211_x_at
GO:0006468	protein phosphorylation	BP	12/1780	2.5245e-8	11740168_at
GO:0005624	membrane fraction	CC	11/1659	1.2746e-7	11742211_x_at
GO:0045261	proton-transporting ATP synthase complex, catalytic core F(1)	CC	3/17	4.1486e-7	11720328_a_at
GO:0042776	mitochondrial ATP synthesis coupled proton transport	BP	3/35	0.0000039496	11720328_a_at
GO:0005488	binding	MF	12/2876	0.0000041965	11742211_x_at
GO:0048488	synaptic vesicle endocytosis	BP	3/40	0.0000059440	11722552_x_at
GO:0046933	hydrogen ion transporting ATP synthase activity, rotational mechanism	MF	3/40	0.0000059440	11720328_a_at
GO:0006200	ATP catabolic process	BP	3/42	0.0000068982	11720328_a_at
GO:0004637	phosphoribosylamine-glycine ligase activity	MF	2/5	0.0000073968	11722930_x_at
GO:0004644	phosphoribosylglycinamide formyltransferase activity	MF	2/5	0.0000073968	11722930_x_at
GO:0004641	phosphoribosylformylglycinamidine cyclo-ligase activity	MF	2/5	0.0000073968	11722930_x_at
GO:0022857	transmembrane transporter activity	MF	3/43	0.0000074111	11720328_a_at
GO:0005753	mitochondrial proton-transporting ATP synthase complex	CC	3/45	0.0000085111	11720328_a_at
GO:0005794	Golgi apparatus	CC	11/2635	0.000011516	11742211_x_at
GO:0005887	integral to plasma membrane	CC	11/2886	0.000026878	11742211_x_at
GO:0006189	'de novo' IMP biosynthetic process	BP	2/11	0.000040547	11722930_x_at
GO:0008159	positive transcription elongation factor activity	MF	2/13	0.000057440	11747292_x_at
GO:0016742	hydroxymethyl-, formyl- and related transferase activity	MF	2/13	0.000057440	11722930_x_at
GO:0009113	purine base biosynthetic process	BP	2/15	0.000077238	11722930_x_at
GO:0015986	ATP synthesis coupled proton transport	BP	3/104	0.00010538	11720328_a_at
GO:0010466	negative regulation of peptidase activity	BP	2/18	0.00011236	11728320_a_at
GO:0005576	extracellular region	CC	12/4296	0.00021890	11742211_x_at
GO:0048513	organ development	BP	2/29	0.00029635	11722930_x_at
GO:0008144	drug binding	MF	3/151	0.00031637	11720328_a_at
GO:0015992	proton transport	BP	3/170	0.00044728	11720328_a_at
GO:0003677	DNA binding	MF	14/6181	0.00051088	11742211_x_at
GO:0006754	ATP biosynthetic process	BP	3/178	0.00051138	11720328_a_at
GO:0006164	purine nucleotide biosynthetic process	BP	2/42	0.00062396	11722930_x_at
GO:0009168	purine ribonucleoside monophosphate biosynthetic process	BP	2/53	0.00099258	11722930_x_at
GO:0035023	regulation of Rho protein signal transduction	BP	3/255	0.0014425	11722552_x_at
GO:0005089	Rho guanyl-nucleotide exchange factor activity	MF	3/257	0.0014751	11722552_x_at
GO:0004869	cysteine-type endopeptidase inhibitor activity	MF	2/70	0.0017233	11728320_a_at
GO:0012505	endomembrane system	CC	3/292	0.0021213	11722552_x_at
GO:0010035	response to inorganic substance	BP	2/85	0.0025265	11722930_x_at
GO:0042375	quinone cofactor metabolic process	BP	1/3	0.0026106	11750534_x_at
GO:0002020	protease binding	MF	2/92	0.0029510	11728320_a_at
GO:0030027	lamellipodium	CC	3/332	0.0030474	11722552_x_at
GO:0005829	cytosol	CC	10/4280	0.0031606	11757031_a_at
GO:0004866	endopeptidase inhibitor activity	MF	2/97	0.0032733	11728320_a_at
GO:0008624	induction of apoptosis by extracellular signals	BP	3/341	0.0032849	11722552_x_at
GO:0003960	NADPH:quinone reductase activity	MF	1/5	0.0043473	11750534_x_at
GO:0005737	cytoplasm	CC	21/14242	0.0043881	11742211_x_at
GO:0016887	ATPase activity	MF	3/387	0.0046754	11720328_a_at
GO:0005085	guanyl-nucleotide exchange factor activity	MF	3/443	0.0067873	11722552_x_at
GO:0043248	proteasome assembly	BP	1/8	0.0069468	11717856_a_at
GO:0005589	collagen type VI	CC	1/9	0.0078118	11715406_a_at
GO:0009058	biosynthetic process	BP	2/155	0.0081272	11722930_x_at
GO:0010033	response to organic substance	BP	2/266	0.022585	11722930_x_at
GO:0046872	metal ion binding	MF	13/8562	0.026746	11742211_x_at
GO:0007051	spindle organization	BP	1/32	0.027506	11723117_a_at
GO:0005743	mitochondrial inner membrane	CC	3/756	0.028052	11720328_a_at
GO:0042623	ATPase activity, coupled	MF	1/37	0.031736	11757031_a_at
GO:0000785	chromatin	CC	2/320	0.031759	11747292_x_at
GO:0005215	transporter activity	MF	3/828	0.035323	11720328_a_at
GO:0000775	chromosome, centromeric region	CC	2/342	0.035852	11724505_at
GO:0048407	platelet-derived growth factor binding	MF	1/44	0.037628	11715406_a_at
GO:0005761	mitochondrial ribosome	CC	1/48	0.040980	11717459_a_at
GO:0042995	cell projection	CC	3/883	0.041473	11722552_x_at
GO:0016925	protein sumoylation	BP	1/51	0.043486	11721797_at
GO:0003702	RNA polymerase II transcription factor activity	MF	2/402	0.047970	11747292_x_at
GO:0016874	ligase activity	MF	3/998	0.055962	11722930_x_at
GO:0016020	membrane	CC	20/16785	0.060228	11739305_a_at
GO:0042384	cilium assembly	BP	1/73	0.061669	11723117_a_at
GO:0044267	cellular protein metabolic process	BP	1/73	0.061669	11757031_a_at
GO:0042981	regulation of apoptosis	BP	2/465	0.062057	11728320_a_at
GO:0008168	methyltransferase activity	MF	2/482	0.066074	11722930_x_at
GO:0070936	protein K48-linked ubiquitination	BP	1/88	0.073873	11731419_x_at
GO:0005815	microtubule organizing center	CC	2/532	0.078373	11757031_a_at
GO:0005515	protein binding	MF	23/20679	0.083287	11742211_x_at
GO:0030433	ER-associated protein catabolic process	BP	1/104	0.086719	11731419_x_at
GO:0050661	NADP or NADPH binding	MF	1/109	0.090698	11750534_x_at
GO:0005814	centriole	CC	1/116	0.096240	11723117_a_at
GO:0007067	mitosis	BP	2/627	0.10349	11724505_at
GO:0030054	cell junction	CC	3/1368	0.11610	11722552_x_at
GO:0043687	post-translational protein modification	BP	1/143	0.11731	11731419_x_at
GO:0019787	small conjugating protein ligase activity	MF	1/153	0.12499	11731419_x_at
GO:0051246	regulation of protein metabolic process	BP	1/155	0.12652	11731419_x_at
GO:0000226	microtubule cytoskeleton organization	BP	1/170	0.13790	11723117_a_at
GO:0005730	nucleolus	CC	4/2320	0.14214	11728320_a_at
GO:0006811	ion transport	BP	3/1609	0.16431	11720328_a_at
GO:0016021	integral to membrane	CC	14/12436	0.17301	11739305_a_at
GO:0051301	cell division	BP	2/885	0.17995	11724505_at
GO:0042383	sarcolemma	CC	1/229	0.18126	11715406_a_at
GO:0005524	ATP binding	MF	6/4420	0.18336	11740168_at
GO:0005739	mitochondrion	CC	5/3498	0.18637	11720328_a_at
GO:0005509	calcium ion binding	MF	3/1779	0.20143	11722552_x_at
GO:0000776	kinetochore	CC	1/259	0.20249	11721797_at
GO:0005882	intermediate filament	CC	1/306	0.23467	11759161_at
GO:0051082	unfolded protein binding	MF	1/347	0.26171	11757031_a_at
GO:0000166	nucleotide binding	MF	7/6002	0.26360	11740168_at
GO:0005813	centrosome	CC	1/437	0.31782	11723117_a_at
GO:0005516	calmodulin binding	MF	1/447	0.32379	11723117_a_at
GO:0006511	ubiquitin-dependent protein catabolic process	BP	1/479	0.34256	11731419_x_at
GO:0006457	protein folding	BP	1/509	0.35969	11757031_a_at
GO:0031012	extracellular matrix	CC	1/538	0.37583	11715406_a_at
GO:0003824	catalytic activity	MF	3/2539	0.38179	11722930_x_at
GO:0005840	ribosome	CC	1/565	0.39051	11717459_a_at
GO:0007049	cell cycle	BP	2/1586	0.40390	11724505_at
GO:0004842	ubiquitin-protein ligase activity	MF	1/613	0.41577	11731419_x_at
GO:0005783	endoplasmic reticulum	CC	3/2848	0.45487	11731419_x_at
GO:0005874	microtubule	CC	1/742	0.47870	11723117_a_at
GO:0005578	proteinaceous extracellular matrix	CC	1/756	0.48511	11715406_a_at
GO:0005792	microsome	CC	1/777	0.49459	11719069_s_at
GO:0005622	intracellular	CC	5/5671	0.55906	11728320_a_at
GO:0030529	ribonucleoprotein complex	CC	1/961	0.57070	11717459_a_at
GO:0005856	cytoskeleton	CC	2/2388	0.62224	11757031_a_at
GO:0005634	nucleus	CC	12/14612	0.65165	11728320_a_at
GO:0004674	protein serine/threonine kinase activity	MF	1/1253	0.66907	11740168_at
GO:0016740	transferase activity	MF	3/4096	0.70372	11740168_at
GO:0005886	plasma membrane	CC	6/7984	0.71582	11720328_a_at
GO:0016491	oxidoreductase activity	MF	1/1572	0.75142	11750534_x_at
GO:0055114	oxidation reduction	BP	1/1592	0.75585	11750534_x_at
GO:0004672	protein kinase activity	MF	1/1623	0.76258	11740168_at
GO:0008152	metabolic process	BP	1/1948	0.82302	11750534_x_at
GO:0006810	transport	BP	3/4997	0.82427	11720328_a_at
GO:0016301	kinase activity	MF	1/2376	0.88018	11740168_at
GO:0007275	multicellular organismal development	BP	1/2597	0.90217	11740168_at
GO:0003700	sequence-specific DNA binding transcription factor activity	MF	1/2801	0.91894	11719541_a_at
GO:0006355	regulation of transcription, DNA-dependent	BP	1/3470	0.95650	11719541_a_at
GO:0007165	signal transduction	BP	1/4684	0.98627	11740168_at
GO:0008270	zinc ion binding	MF	1/5672	0.99475	11750534_x_at
GO:0006350	transcription	BP	1/6068	0.99645	11719541_a_at
GO:0045449	regulation of transcription	BP	1/6260	0.99707	11719541_a_at
