# Supplementary material for: Interactive knowledge discovery and data mining on genomic expression data with numeric formal concept analysis
Source: BMC Bioinformatics. 2016 Sep 15;17:374. doi: 10.1186/s12859-016-1234-z (PMC5024470; doi:10.1186/s12859-016-1234-z)
Supplement: Additional file 5 — Print-out of GO terms’ information for gene enrichment from WebGeneKFCA A sample print-out from WebGeneKFCA with the information about the GO terms and their p-values computed as described in http://www.biomedcentral.com/content/supplementary/10.1186/s12859-016-1234-z-S2.pdfAdditional file 2 for the bicluster with intent MalePS, Parental NoDox, Clone 1 NoDox and Clone 3 NoDox from Fig. 4. It can also be obtained on-line at https://webgenekfca.com/webgenekfca/kfcaresultses/9 as a CSV file. (PDF 358 kb) [file 12859_2016_1234_MOESM5_ESM.pdf]

This view shows a full lattice with all the possible concepts for all the possible microarrays experiments available in the gene expression matrix. In the lattice the blue circles are the concepts where the selected gene belongs for a ϕ threshold range, the blue lines show the path that follows the selected gene between the different concepts when the threshold changes. The radius of each concept is proportional to the threshold range where the probeset remains in that concept, this means that a concept will be larger if the selected probeset remains longer on that concept when sweeping over ϕ.

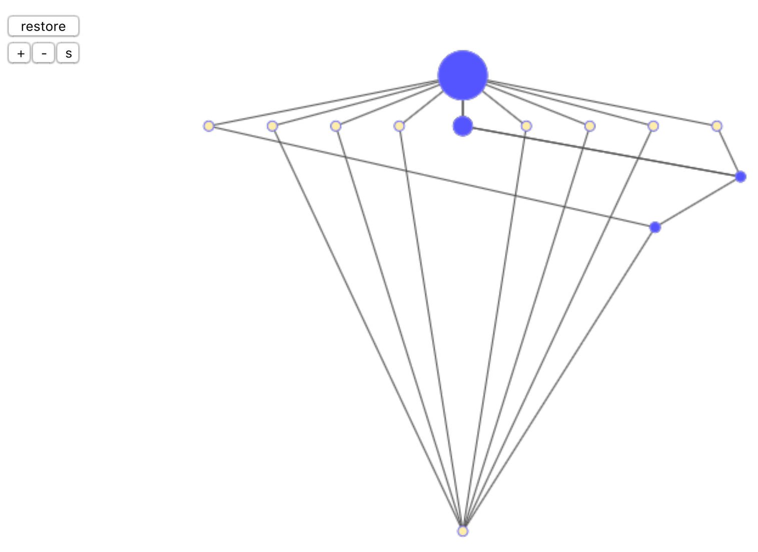

Probe Set ID	11742211_x_at
Gene Title	amyloid beta (A4) precursor protein
Target Description	gb:AK294534.1 /DB_XREF=gi:194377585 /TID=Hs.434980.3 /CNT=2 /FEA=FLmRNA /TIER=FL /STK=1 /LL=351 /UG_GENE=APP /UG=Hs.434980 /UG_TITLE=Amyloid beta (A4) precursor protein /DEF=Homo sapiens cDNA FLJ50491 complete cds, highly similar to Amyloid beta A4 protein precursor (APP) (ABPP)(Alzheimer disease amyloid protein) (Cerebral vascularamyloid peptide) (CVAP) (Protease nexin-II) (PN-II)(APPI) (PreA4) (Contains: Soluble APP-alpha (S-APP-alpha); Soluble APP-beta (S-APP-beta); C99; Beta-amyloidprotein 42 (Beta-APP42); Beta-amyloid protein 40(Beta-APP40); C83; P3(42); P3(40); Gamma-CTF(59)(Gamma-secretase C-terminal fragment 59) (Amyloidintracellular domain 59) (AID(59)); Gamma-CTF(57)(Gamma- secretase C-terminal fragment 57) (Amyloidintracellular domain 57) (AID(57)); Gamma-CTF(50)(Gamma-secretase C-terminal fragment 50) (Amyloidintracellular domain 50) (AID(50)); C31). /FL=gb:AK294534.1 gb:NM_001136130.2 /REP_ORG=H. sapiens
AGI	
Gene Symbol	APP
QTL	

Gene Ontology Biological Process

Accession	Definition
GO:0000085	G2 phase of mitotic cell cycle
GO:0000085	G2 phase of mitotic cell cycle
GO:0001967	suckling behavior
GO:0006378	mRNA polyadenylation
GO:0006378	mRNA polyadenylation
GO:0006417	regulation of translation
GO:0006417	regulation of translation
GO:0006468	protein phosphorylation
GO:0006468	protein phosphorylation
GO:0006878	cellular copper ion homeostasis
GO:0006878	cellular copper ion homeostasis
GO:0006897	endocytosis
GO:0006897	endocytosis
GO:0006915	apoptosis
GO:0006917	induction of apoptosis
GO:0007155	cell adhesion
GO:0007176	regulation of epidermal growth factor receptor activity
GO:0007176	regulation of epidermal growth factor receptor activity
GO:0007219	Notch signaling pathway
GO:0007409	axonogenesis
GO:0007409	axonogenesis
GO:0007617	mating behavior
GO:0007617	mating behavior
GO:0007626	locomotory behavior
GO:0007626	locomotory behavior
GO:0008088	axon cargo transport
GO:0008088	axon cargo transport
GO:0008219	cell death
GO:0008344	adult locomotory behavior
GO:0008344	adult locomotory behavior
GO:0008542	visual learning
GO:0008542	visual learning
GO:0016199	axon midline choice point recognition
GO:0016199	axon midline choice point recognition
GO:0016322	neuron remodeling
GO:0016322	neuron remodeling
GO:0016358	dendrite development
GO:0016358	dendrite development
GO:0030198	extracellular matrix organization
GO:0030198	extracellular matrix organization
GO:0030900	forebrain development
GO:0031175	neuron projection development
GO:0031175	neuron projection development
GO:0035235	ionotropic glutamate receptor signaling pathway
GO:0035235	ionotropic glutamate receptor signaling pathway
GO:0040014	regulation of multicellular organism growth
GO:0040014	regulation of multicellular organism growth
GO:0045931	positive regulation of mitotic cell cycle
GO:0045931	positive regulation of mitotic cell cycle
GO:0045944	positive regulation of transcription from RNA polymerase II promoter
GO:0048669	collateral sprouting in absence of injury
GO:0048669	collateral sprouting in absence of injury
GO:0050803	regulation of synapse structure and activity
GO:0050803	regulation of synapse structure and activity
GO:0050885	neuromuscular process controlling balance
GO:0051124	synaptic growth at neuromuscular junction
GO:0051402	neuron apoptosis
GO:0051563	smooth endoplasmic reticulum calcium ion homeostasis

Gene Ontology Cellular Component

Accession	Definition
GO:0005576	extracellular region
GO:0005624	membrane fraction
GO:0005737	cytoplasm
GO:0005737	cytoplasm
GO:0005737	cytoplasm
GO:0005794	Golgi apparatus
GO:0005794	Golgi apparatus
GO:0005794	Golgi apparatus
GO:0005887	integral to plasma membrane
GO:0005905	coated pit
GO:0009986	cell surface
GO:0016020	membrane
GO:0016021	integral to membrane
GO:0016021	integral to membrane
GO:0019717	synaptosome
GO:0030424	axon
GO:0030424	axon
GO:0031410	cytoplasmic vesicle
GO:0031594	neuromuscular junction
GO:0035253	ciliary rootlet
GO:0043005	neuron projection
GO:0043197	dendritic spine
GO:0043198	dendritic shaft
GO:0045177	apical part of cell
GO:0045202	synapse
GO:0048471	perinuclear region of cytoplasm
GO:0051233	spindle midzone

Gene Ontology Molecular Function

Accession	Definition
GO:0003677	DNA binding
GO:0003677	DNA binding
GO:0004867	serine-type endopeptidase inhibitor activity
GO:0004867	serine-type endopeptidase inhibitor activity
GO:0005102	receptor binding
GO:0005488	binding
GO:0005515	protein binding
GO:0005515	protein binding
GO:0008201	heparin binding
GO:0008233	peptidase activity
GO:0016504	peptidase activator activity
GO:0030414	peptidase inhibitor activity
GO:0033130	acetylcholine receptor binding
GO:0042802	identical protein binding
GO:0046872	metal ion binding

Pathway	---
GeneChip Array	
Transcript ID(Array Design)	Hs.434980.3
Species Scientific Name	
Annotation Date	Aug 25, 2011
Sequence Type	Consensus sequence
Sequence Source	GenBank
Representative Public ID	AK294534.1
Archival UniGene Cluster	Hs.434980
UniGene ID	Hs.434980
Genome Version	February 2009 (Genome Reference Consortium GRCh37)
Alignments	chr21:27252861-27543138 (-) // 94.54 // q21.3
Gene Symbol	APP
Chromosomal Location	chr21q21.2|21q21.3
Unigene Cluster Type	full length
Ensembl	ENSG00000142192
Entrez Gene	351
SwissProt	null
EC	---
OMIM	104300 /// 104760 /// 605714
RefSeq Protein ID	
RefSeq Transcript ID	
FlyBase	---
WormBase	
MGI Name	---
RGD Name	---
SGD accession number	---
InterPro	
Trans Membrane	
Annotation Description	
Annotation Transcript Cluster	
